# SeqEnrich: A tool to predict transcription factor networks from co-expressed Arabidopsis and *Brassica napus* gene sets

**DOI:** 10.1371/journal.pone.0178256

**Published:** 2017-06-02

**Authors:** Michael G. Becker, Philip L. Walker, Nadège C. Pulgar-Vidal, Mark F. Belmonte

**Affiliations:** 1 Department of Biological Sciences, University of Manitoba, Winnipeg, Manitoba, Canada; 2 Department of Computer Science, University of Manitoba, Winnipeg, Manitoba, Canada; McGill University, CANADA

## Abstract

Transcription factors and their associated DNA binding sites are key regulatory elements of cellular differentiation, development, and environmental response. New tools that predict transcriptional regulation of biological processes are valuable to researchers studying both model and emerging-model plant systems. SeqEnrich predicts transcription factor networks from co-expressed Arabidopsis or *Brassica napus* gene sets. The networks produced by SeqEnrich are supported by existing literature and predicted transcription factor–DNA interactions that can be functionally validated at the laboratory bench. The program functions with gene sets of varying sizes and derived from diverse tissues and environmental treatments. SeqEnrich presents as a powerful predictive framework for the analysis of Arabidopsis and *Brassica napus* co-expression data, and is designed so that researchers at all levels can easily access and interpret predicted transcriptional circuits. The program outperformed its ancestral program ChipEnrich, and produced detailed transcription factor networks from Arabidopsis and *Brassica napus* gene expression data. The SeqEnrich program is ideal for generating new hypotheses and distilling biological information from large-scale expression data.

## Introduction

Advances in next generation RNA sequencing (RNA-seq) technologies to investigate biological processes is becoming commonplace in research laboratories. Despite these advances, the analyses of large scale RNA-seq datasets are time consuming and publicly available tools able to analyze this information are often difficult to navigate for researchers with limited bioinformatics experience. Deriving relevant biological information from large-scale datasets represents a significant bottleneck in sequencing experiments. Thus, new user-friendly tools that facilitate analyses of large datasets are in demand from scientists studying both model and emerging-model plant systems.

A major objective in gene expression analyses is the identification of transcription factors (TFs) and cis-regulatory elements that direct cellular bioprocesses at the cellular, tissue, or whole plant level or in response to biotic or abiotic stresses. Many of the currently available tools, including Grassius Regulatory Grid Explorer hosted by the Arabidopsis Gene Regulatory Information Server (AGRIS; [1]) and HRGRN [2], are ideal for individual gene lookups but are not conducive to large datasets. Additionally, several tools can effectively identify enriched promoter motifs from Arabidopsis datasets, such as the motif analysis tool hosted by The Arabidopsis Information Server (TAIR; [3]), the Cistome tool at the Toronto the Bio-Analytic Resource (BAR; [4]), and Arabidopsis Motif Scanner [5]; however, these programs are not designed to identify enriched biological processes or generate TF networks. The Arabidopsis Interactions Viewer hosted by the Toronto BAR identifies experimentally validated protein-DNA interactions and generates a transcription network from an input query list [6]. This tool can build on this network by adding predicted or validated protein-protein interactions. Ultimately, this provides detailed and accurate information on direct interactions between genes within a dataset, but reliance on experimentally validated protein-DNA interactions limits discovery. Further, the Arabidopsis Interactions Viewer does not identify enriched cis-regulatory elements or relate networks back to biological function. Further, none of the programs above take input data derived from emerging model species *Brassica napus* (canola).

ChipEnrich was developed to perform promoter analysis, TF-DNA binding prediction, and functional enrichment in a single Java-based tool [7,8]. Developed to analyze large-scale co-expressed gene sets from the Arabidopsis ATH1 microarray, ChipEnrich takes a list of genes identified through clustering or differential gene expression analyses and identifies enriched Gene Ontology (GO) terms, TF families, and TF binding site motifs. The program also offers an analysis function that associates these terms into a TF network thus providing a predictive framework into the transcriptional programs of co-expressed gene sets. While this program has been used successfully in the analysis of large-scale microarray data [8–10], it is limited to ATH1 GeneChip sequences of the model plant Arabidopsis and publicly available information on i) gene function ii) nucleotide sequence of DNA binding sites and iii) TF-DNA sequence motif interactions.

Here, we developed SeqEnrich (S1 File) based on the ChipEnrich platform to predict TF networks from next generation RNA-seq datasets. The SeqEnrich program contains the most extensive database of TFs, TF-DNA sequence motif interactions, and gene function(s) for the efficient interrogation of Arabidopsis or *B*. *napus* gene sets. SeqEnrich was able to successfully predict TF networks supported by existing experimental data in addition to providing new insights into the underlying transcriptional circuitry controlling biological process in space and time. This program demonstrates a substantial improvement when compared to its ancestor ChipEnrich. SeqEnrich complements existing tools available for Arabidopsis, merging the functions of multiple tools into a single user-friendly program. Additionally, this serves as the first resource able to produce transcription factor networks from *B*. *napus* datasets.

## Materials and methods

### Program execution

The SeqEnrich software was developed using Java language and designed to be fully compatible with Windows and Linux operating systems. SeqEnrich is a user-friendly application that takes an input text format (.txt) query list of Arabidopsis Genome Initiative [11] identifiers or *B*. *napus* annotation v5 identifiers [12]. Input gene lists are developed through differential gene expression or clustering analyses performed following next generation RNA-seq experiments.

The SeqEnrich jar file (S1 File) and SeqEnrich source code (S2 File) are freely available. Updated versions of the SeqEnrich program will be deposited as they become available at http://www.belmontelab.com and updated source code deposited at the SourceForge open-source repository (https://sourceforge.net/).

### GO term enrichment

GO term enrichment was defined as the ratio between (a) the number of genes in the query list belonging to the GO term and (b) the total number of genes belonging to the GO term within the genome compared to the ratio of (c) the total number of genes within the query list to (d) the total number of genes within the genome. Significantly enriched GO terms are reported as p-values calculated from the hypergeometric distribution using the Apache Commons Math Library (http://jakarta.apache.org/commons/math). SeqEnrich reports all GO term enrichment data and produces a summary file containing significantly enriched data with a p-value of < 0.001.

### DNA sequence motif enrichment

For each gene within the input query list, known TF binding sites are called from a lookup array within the program database. This array was created by identifying known TF binding sites within all gene promoters in the *B*. *napus* and Arabidopsis genomes. Here, we focus on motif enrichment within the 1 kb upstream region from the transcription start site, capturing the majority of Arabidopsis TF binding sites [13]. Background motif distributions are determined from the TF binding sites identified within all promoters across the genome. Statistical enrichment for each DNA sequence motif within the query list was determined using the hypergeometric distribution. In addition, a subanalysis identifies DNA sequence motifs enriched within promoters of query genes associated with each enriched GO term. SeqEnrich reports all motif enrichment data and produces a summary file containing significantly enriched motifs with a p-value of < 0.001.

### Prediction of transcription factor networks

**Fig 1:**
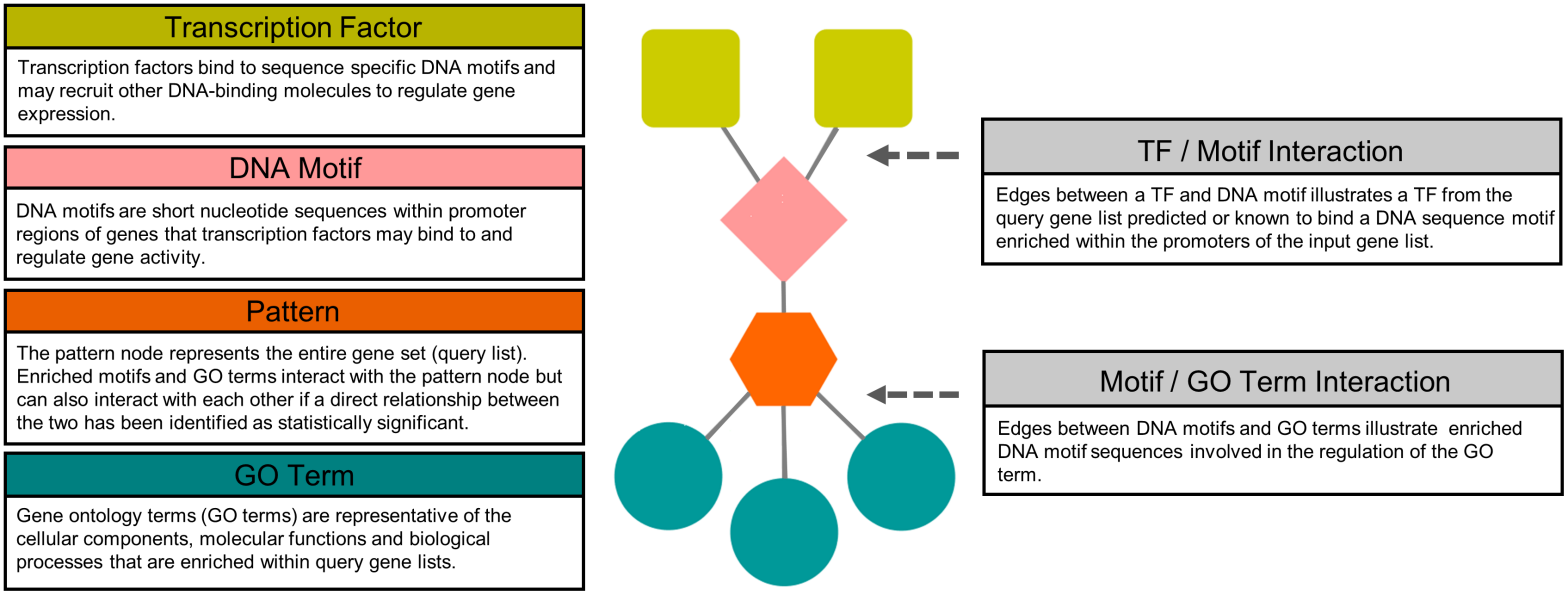
Conceptual description of a transcription factor network. TFs are represented as green rounded squares, DNA motifs as pink diamonds, gene patterns as orange hexagons, and gene ontology (GO) terms as blue circles. Connections between TFs and motifs, and between motifs and patterns/GO terms are represented by a grey connecting line.

Fig 1 shows a conceptual TF network generated using the SeqEnrich program. To produce TF networks, the SeqEnrich program performs an analysis and subanalysis of the input query list. In the analysis, DNA sequence motifs significantly (p < 0.001) enriched in promoters of query genes are associated with TFs within the same query gene list capable of binding to that DNA sequence motif. In the subanalysis, DNA sequence motifs significantly (p < 0.001) enriched in promoters of query genes of individual significantly enriched GO terms (p < 0.001) are associated with TFs within the query gene list capable of binding to that sequence motif. Together, these analyses produce connections between TFs, enriched DNA motifs, query genes (gene pattern), and GO terms and is presented in output network files. SeqEnrich produces two network files: i) TF networks from the analysis identifying DNA sequence motifs and GO terms enriched within the entire query list, and the associated TFs (extension.analysis.networks.txt) and ii) TF networks from the subanalysis identifying motifs enriched within each individual enriched GO term, and associated TFs (extension.subanalysis.networks.txt). Output network files are compatible with the Cytoscape visualization tool (http://www.cytoscape.org/), and p-values imported as edge attributes. A separate attributes file is produced and labels each node presented in the network file as a “pattern”, “GO term”, “motif”, or “TF”.

### Database construction

**Fig 2:**
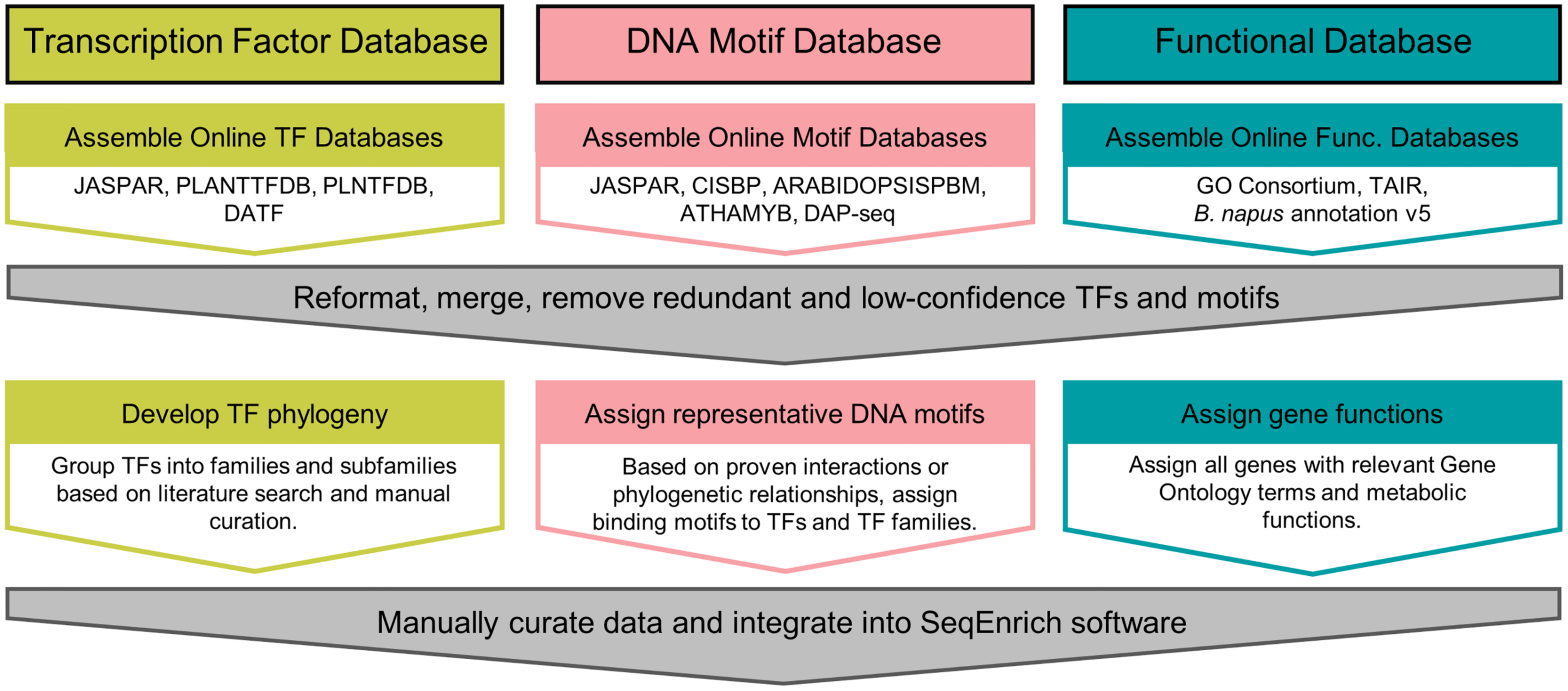
Design and assembly of the SeqEnrich database. Information on transcription factors, DNA binding site motifs, and gene functions were collected from publically available sources and integrated into the SeqEnrich program.

The database for the SeqEnrich program was built using information from publicly available sources (Fig 2). The GO term database and genome annotations were derived from data curated by the TAIR consortium (TAIR10, https://www.arabidopsis.org) and *B*. *napus* annotation v5 [12]. Arabidopsis and *B*. *napus* TFs were identified using information from several databases to ensure inclusion of all TFs: i) JASPAR (http://jaspar.genereg.net/) ii) Plant TFDB (http://planttfdb.cbi.pku.edu.cn/) iii) Plant Transcription Factor Database (http://plntfdb.bio.uni-potsdam.de/) and iv) Database of Arabidopsis Transcription Factors (http://datf.cbi.pku.edu.cn/). A database of experimentally validated TF binding sites was built using information from the following sources: i) JASPAR ii) CIS-BP (http://cisbp.ccbr.utoronto.ca/) iii) Arabidopsis protein binding microarray [14] iv) Yeast-one hybrid experiments [15] and v) Arabidopsis DAP-sequencing experiments [16].

All motif databases presenting motifs in MEME format were converted into degenerate IUPAC codes using a custom Java script (S3 File) to ensure consistency and clarity amongst motifs. If the probability of any given nucleotide at each position within a motif is <0.10, it is not recorded at that position during generation of the IUPAC code (Table 1). This probability cut-off provided an ideal balance between specificity and sensitivity. Lower thresholds that were tested generated overly general motifs, and higher thresholds eliminated putative binding sites.

**Table 1:** IUPAC codes used for representation of nucleotides in motifs and corresponding likelihood of each nucleotide at position.

IUPAC code	IUPAC identity	Nucleotide probabilities
**A**	Adenine	[p(A) ≥ 0.1, p(T) < 0.1, p(C) < 0.1, p(G) < 0.1]
**C**	Cytosine	[p(A) < 0.1, p(T) < 0.1, p(C) ≥ 0.1, p(G) < 0.1]
**G**	Guanine	[p(A) < 0.1, p(T) < 0.1, p(C) < 0.1, p(G) ≥ 0.1]
**T**	Thymine	[p(A) < 0.1, p(T) ≥ 0.1, p(C) < 0.1, p(G) < 0.1]
**R**	A or G	[p(A) ≥ 0.1, p(T) < 0.1, p(C) < 0.1, p(G) ≥ 0.1]
**Y**	C or T	[p(A) < 0.1, p(T) ≥ 0.1, p(C) ≥ 0.1, p(G) < 0.1]
**S**	G or C	[p(A) < 0.1, p(T) < 0.1, p(C) ≥ 0.1, p(G) ≥ 0.1]
**W**	A or T	[p(A) ≥ 0.1, p(T) ≥ 0.1, p(C) < 0.1, p(G) < 0.1]
**K**	G or T	[p(A) < 0.1, p(T) ≥ 0.1, p(C) < 0.1, p(G) ≥ 0.1]
**M**	A or C	[p(A) ≥ 0.1, p(T) < 0.1, p(C) ≥ 0.1, p(G) < 0.1]
**B**	C or G or T	[p(A) < 0.1, p(T) ≥ 0.1, p(C) ≥ 0.1, p(G) ≥ 0.1]
**D**	A or G or T	[p(A) ≥ 0.1, p(T) ≥ 0.1, p(C) < 0.1, p(G) ≥ 0.1]
**H**	A or C or T	[p(A) ≥ 0.1, p(T) ≥ 0.1, p(C) ≥ 0.1, p(G) < 0.1]
**V**	A or C or G	[p(A) ≥ 0.1, p(T) < 0.1, p(C) ≥ 0.1, p(G) ≥ 0.1]

Following amalgamation of motif databases, data filtering was used to remove duplicate entries and non-informative motifs. A non-informative motif was defined as a motif that lacked sequence specificity and had a p < 0.02 of occurring at any individual locus within a promoter by chance. To improve the resolution and accuracy of SeqEnrich, TF phylogenetic relationships were used to predict TF-DNA sequence motif interactions. For example, TFs with no experimentally validated binding information or TFs with conflicting experimental data could be assigned putative DNA binding site motifs based on information from closely related TFs. These TF phylogenetic relationships were determined by combining existing TFF phylogenetic footprinting data [15,17–26] and protein clustering performed with MUSCLE v3.8.31 (http://www.drive5.com/muscle/muscle.html). Using TF phylogenetic relationships to predict TF binding sites represents a new feature of SeqEnrich and dramatically improves the number of TFs with known or predicted binding sites, with 902 new predicted TF-DNA sequence motif interactions as compared to the original ChipEnrich program.

## Results and discussion

### The SeqEnrich database

The SeqEnrich database contains a total of 2,263 Arabidopsis TFs and 240 Arabidopsis degenerate DNA binding site motifs, representing a 44.8% and 135% increase respectively, as compared to the original ChipEnrich software. As an additional comparison, the AGRIS databases [1] currently contain 1,773 TFs and 99 DNA binding site motifs (http://arabidopsis.med.ohio-state.edu). The majority (85.4%) of TFs in the AGRIS transcription factor database overlapped with SeqEnrich. For the remaining ~15%, many were incorrectly classified as C3H family TFs due to their zinc finger, RING-type domains. Experimental evidence has suggested that they are more likely involved in ubiquitination [27,28]. The newly developed *B*. *napus* database contains 8,306 TFs and 228 degenerate motifs. The greater number of TFs in *B*. *napus* can be explained by a genome duplication event since its divergence from Arabidopsis and its large allotetraploid genome of ~90,000 genes [12]. The decline in the number of binding site motifs identified in *B*. *napus* is likely due to its comparative lack of experimental TF binding data.

Since little information about *B*. *napus* TF binding sites are available, the majority of *B*. *napus* binding site motifs are predicted from closely related Arabidopsis homologs. Using homology to predict TF binding sites is common in mammalian systems [29,30], and is supported by multiple independent experiments that identify conserved TF target sequences between plant species as distally related as Arabidopsis and rice [21,22,31]. Our SeqEnrich program represents the largest database for *B*. *napus* TFs and motifs currently available, and is accessible within the program source code (S2 File) and as a separate download at http://www.belmontelab.com.

### Validation of the SeqEnrich program

**Fig 3:**
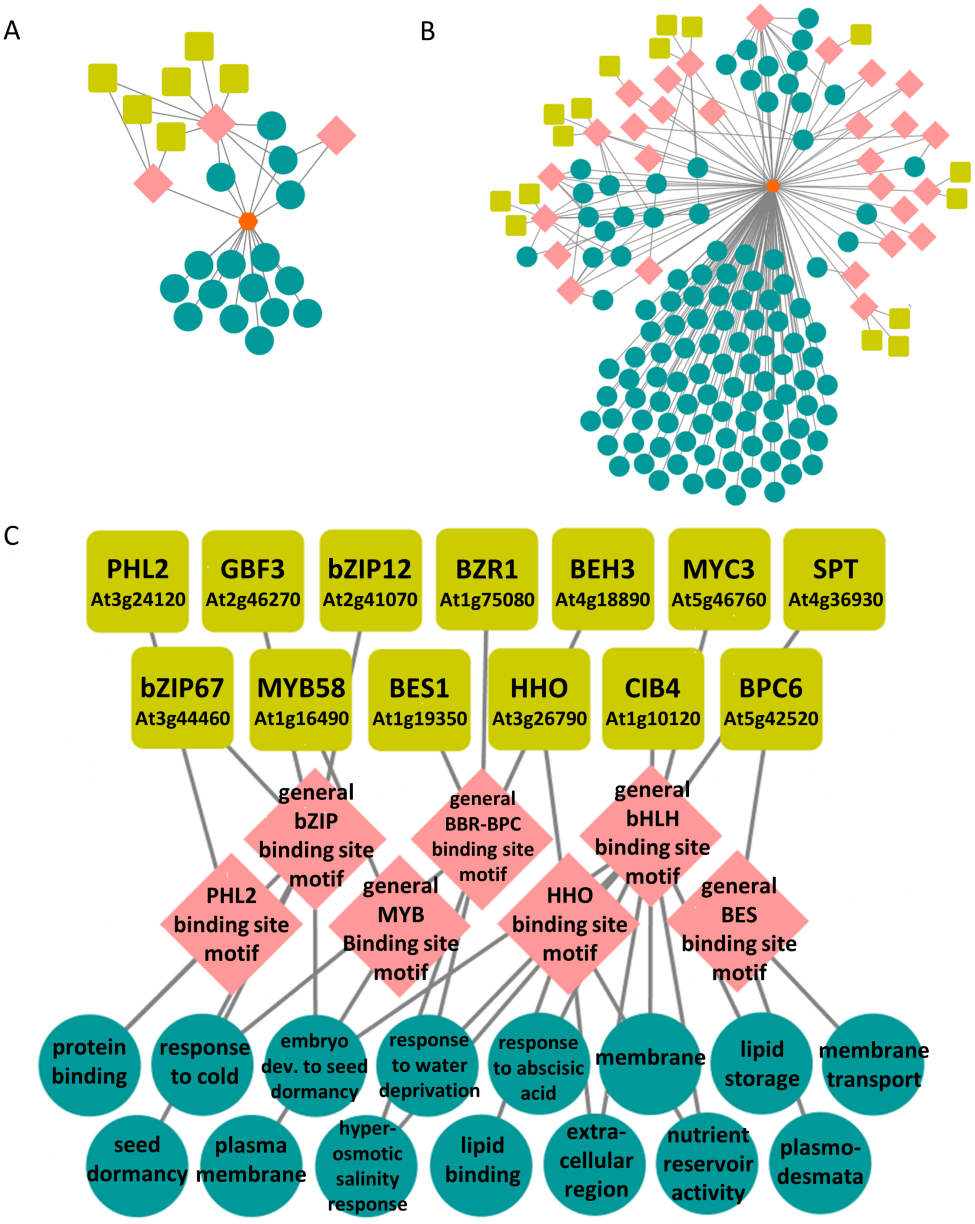
Predicted transcription factor networks from the chalazal endosperm of Arabidopsis. A) Predicted transcriptional module developed from the ChipEnrich program; B) predicted transcriptional module using the SeqEnrich program; C) subset of the transcriptional module produced from the subanalysis function of the SeqEnrich program. A predicted bZIP, bHLH, MYB, and BES transcriptional module controlling biological processes within the mature endosperm of Arabidopsis.

To demonstrate how the SeqEnrich program improves detection of TF-DNA sequence motif interactions, we compared its output to previously published Arabidopsis TF networks from publicly available datasets [8,32]. A total of 661 genes co-expressed in the chalazal endosperm of the mature seed (S4 File) were used as input into both the ChipEnrich (Fig 3A) and SeqEnrich programs (Fig 3B). This comparison identified 2.6-fold more TFs, 7.1-fold more enriched GO terms, and 9.3-fold more enriched motifs using the SeqEnrich program with the same gene query list. As the SeqEnrich database contains only 44.8% more motifs than ChipEnrich, the observed increase in motif detection is likely due to the improved accuracy of new databases and not solely a result of increase in database size. Similarly, the larger number of enriched GO terms in SeqEnrich can be attributed to the improvements in TAIR curated GO annotations.

We also identified predictive TF networks in mature chalazal endosperm using the subanalysis function of SeqEnrich (Fig 3C). This transcriptional circuit predicts the regulation of a number of seed-related bioprocesses, including “nutrient reservoir activity”, “embryo development to seed dormancy”, “lipid storage”, “response to abscisic acid”, and “seed dormancy” by TFs binding to the BZIP, MYB, BES, and BHLH binding sites. As the subanalysis function is a new function in the program a direct comparison between subanalyses is not possible; however, several of the DNA binding sites identified, including PHL2, BBR-BPC, BES, HHO are found exclusively within the SeqEnrich database. Several of these TFs identified in this circuit, including SPATULA and BZR1, are already associated with seed maturation and have delayed development phenotypes [33,34]. Other TFs identified in the subanalysis, including PHL2, CIB4, and BPC6, have no known biological function. Based on the late timing of their expression, they may serve as transcriptional regulators of seed maturation active in tissue-specific subregions of the seed.

To confirm that the SeqEnrich output is reflective of *in vivo* gene regulation, we tested the program on a dataset containing TFs and DNA targets with proven interactions. We used the Taylor-Teeples et al. (2015) dataset of coexpressed genes involved in secondary cell wall biosynthesis of the Arabidopsis steele, which includes TF-motif interactions confirmed with yeast one hybrid experiments [32]. We produced a network with these data containing 58 TFs targeting genes associated with cell wall, xylem, lignin, cellulose and hemicellulose, root development, and regulation of transcription (S1 Fig). Our analysis identified enriched bZIP, homeobox domain (HD), and zinc-finger HD transcription factor families, which agrees with the findings in Taylor-Teeples et al. (2015). We also identified TFs PHABULOSA and REVOLUTA in our networks, which were a center of focus in Taylor-Teeples et al. (2015) and proven central regulators of xylem cell specification and secondary cell wall biosynthesis. Together, these data support the ability of SeqEnrich to report meaningful biological predictions.

### SeqEnrich predicts transcription factor networks in *Brassica napus*

**Fig 4:**
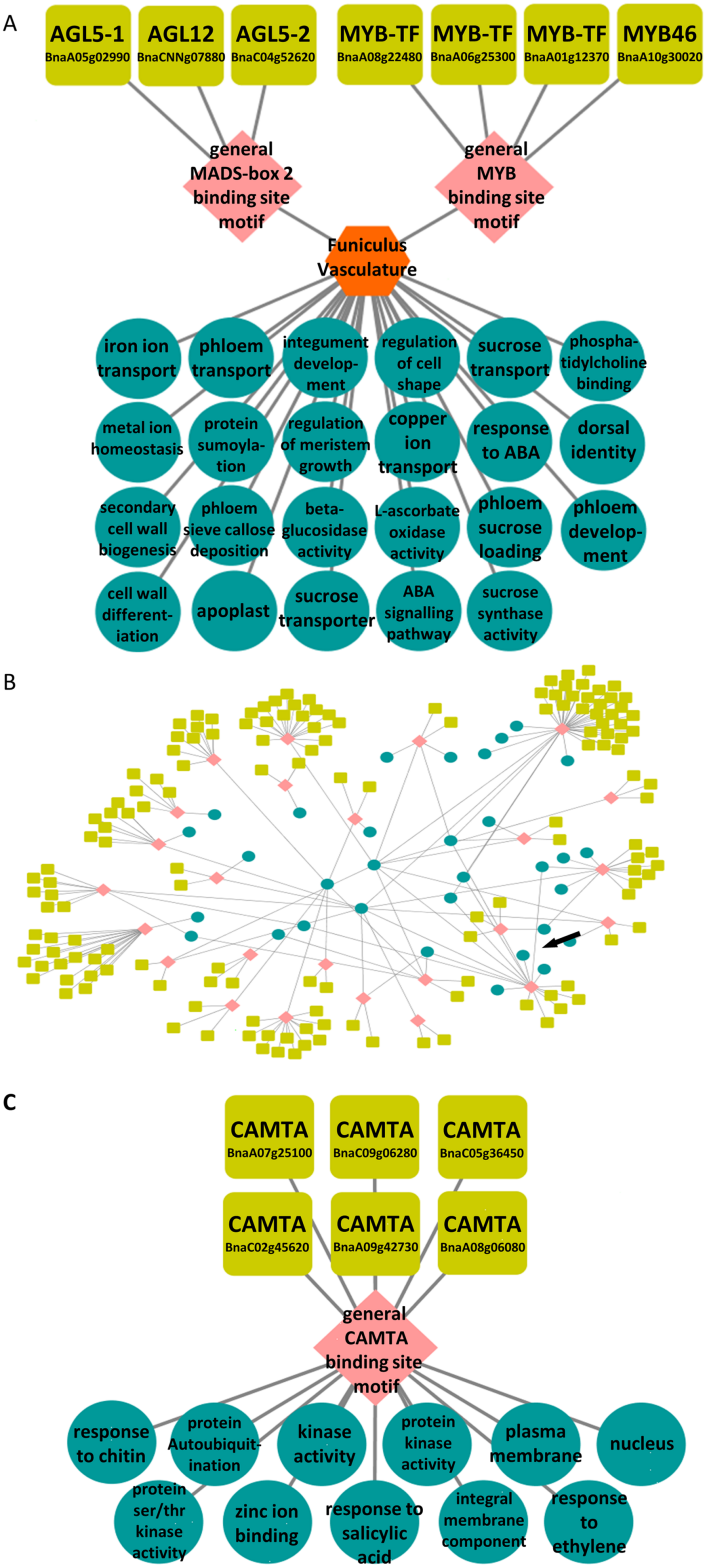
Predicted transcription factor networks from *Brassica napus* gene sets. A) Subset of transcription factor network identified from funiculus vasculature dataset. MYB and MADS-box TFs are predicted to regulate genes associated with transport, metal ion homeostasis, and cell wall modification; B) subset of transcriptional module identified from the SeqEnrich subanalysis function predicted to be operative in seedlings infected with fungal pathogen *Leptosphaeria maculans*; C) transcriptional module depicted by arrow in (B), showing regulation of genes associated with defense bioprocesses by a family of calmodulin-binding transcriptional activators (CAMTAs).

We used two different datasets (S4 File) to test the efficacy of the SeqEnrich program on large-scale *B*. *napus* RNA-seq co-expressed gene lists: i) a tissue-specific dataset of 684 genes activated within the vasculature of the *B*. *napus* funiculus [35] (Fig 4A); and ii) a defense response dataset of 3,234 genes activated specifically in cotyledons resistant to the fungal pathogen *Leptosphaeria maculans* [36] (Fig 4B–4C). These datasets were selected because of i) the differences in the size of the query list, ii) the tissue type from where RNA sequencing was performed, and iii) the environmental conditions in which the plants were reared based on their cellular response.

The newly developed SeqEnrich program identified TFs binding to the MYB and MADS-box binding site motifs enriched within the promoters of genes specific to funiculus vasculature, and are associated with “phloem sieve callose deposition”, “secondary cell wall biogenesis”, “phloem sucrose loading”, “integument development”, and “metal ion homeostasis” (Fig 4A). As the only connection between maternal tissue and the growing seed, the funiculus is responsible for nutrient transfer during seed filling [35]. Two of the TFs identified in this transcriptional circuit have proven roles in plant root tissue [37,38], which may be due to the similar functions of root and funicular tissues in nutrient transport [39]. For example, MYB46 is concentrated within the xylem of growing roots and a master regulator of secondary cell wall formation [37]. The identification of MYB46 in funicular vasculature (Fig 4A) clearly demonstrates the ability of SeqEnrich to generate new scientific hypotheses that can be functionally validated at the bench.

The SeqEnrich subanalysis predicted a large transcriptional network active in resistant *B*. *napus* cotyledons, suggesting that coordination of the defense response is complex and involves large numbers of TFs to control expression of genes responsible for plant defense and highlights the sensitivity of the program (Fig 4B). Most TFs identified converged on similar binding site motifs. For example, 30 WRKY TFs were identified in a transcriptional module and are predicted to bind to a similar WRKY binding site motif. The large number of cognate TFs is likely due to gene duplication in the *B*. *napus* genome and redundant TF functions. A subsection of this network is presented in Fig 4C and predicts a family of calmodulin binding transcriptional activators (CAMTAs) binding to the CAMTA binding site motif enriched in gene promoters associated with “response to chitin”, “kinase activity”, “response to salicylic acid”, and “response to ethylene” during defense. As calmodulin has been shown to accumulate in resistant plants upon pathogen exposure [40], the activity of calmodulin-binding TFs is logical and provides additional evidence into the predictive framework of the SeqEnrich analysis using *B*. *napus* data. As the binding sites of these CAMTAs were unknown until recent DAP-seq experiments [16] this clearly demonstrates the sensitivity of our newly developed program afforded by its carefully constructed DNA sequence motif databases.

### Program utility

Tools to identify enriched GO terms [41] or DNA sequence motifs [5,42–44] from Arabidopsis gene sets are available, however, none exist that combine these functions into a complete analysis that identifies TF-DNA sequence interactions within biological processes encoded by gene sets of RNA-seq data. Here, SeqEnrich identifies enriched DNA sequence motifs within the promoters of query gene sets or a smaller subset of genes belonging to a biological process with predicted or known TF-DNA sequence motif interactions. Further, the SeqEnrich program interrogates current databases of GO terms, DNA sequence motifs, and TFs thus reducing manual data curation by the user. Current user-friendly programs that facilitate rapid analysis of RNA-seq data and elucidation of regulatory networks are limited to humans and other mammalian systems [45–48]. Our SeqEnrich program serves the plant biology community and is designed for the model organism Arabidopsis and economically important *B*. *napus*. Given the utility and applications of the program and the continued generation of large scale RNA-seq datasets from model and emerging model plant systems, we are currently developing similar data analysis platforms for globally-important monocot crop systems such as rice and corn. The hypotheses generated from this program can then be transferred to the laboratory bench to functionally characterize the underlying molecular mechanisms of the plant.

## Conclusion

The SeqEnrich program represents a powerful user-friendly platform for the analyses of large scale RNA-seq datasets and the prediction of TF networks from differential gene expression or co-expression analyses. We show the program can be applied broadly across query gene lists of different sizes, tissue types, and experimental treatments. The program’s ease of use allows researchers without a background in computational biology to perform comprehensive analysis of RNA-seq data and identify TF-DNA motif interactions orchestrating complex biological processes.

## Supporting information

S1 File SeqEnrich is a Java based program and fully compatible with Windows or Linux operating systems. Detailed user instructions are provided with the SeqEnrich file. Updated versions of the SeqEnrich program will be deposited as they become available at http://www.belmontelab.com.
(ZIP)

S2 File SeqEnrich source code. Updated versions of the SeqEnrich source code will be deposited as they become available at the SourceForge open-source repository (https://sourceforge.net/).
(ZIP)

S3 File File contains both the Java file and usage information. (ZIP)

S4 File Arabidopsis and *B*. *napus* query lists used as test data for the SeqEnrich program. (ZIP)

S1 Fig Transcription factor network from experimentally validated TF-DNA interactions from a root xylem secondary cell wall biosynthesis dataset. (TIFF)
