# Supplementary material for: SeqEnrich: A tool to predict transcription factor networks from co-expressed Arabidopsis and Brassica napus gene sets
Source: PLoS One. 2017 Jun 2;12(6):e0178256. doi: 10.1371/journal.pone.0178256 (PMC5456048; doi:10.1371/journal.pone.0178256)
Supplement: S1 File — Detailed user instructions are provided with the SeqEnrich file. Updated versions of the SeqEnrich program will be deposited as they become available at http://www.belmontelab.com. (ZIP) [file pone.0178256.s001.zip › SeqEnrich/SeqEnrich-Instructions.docx]

**Building predictive transcription factor networks with SeqEnrich and Cytoscape**


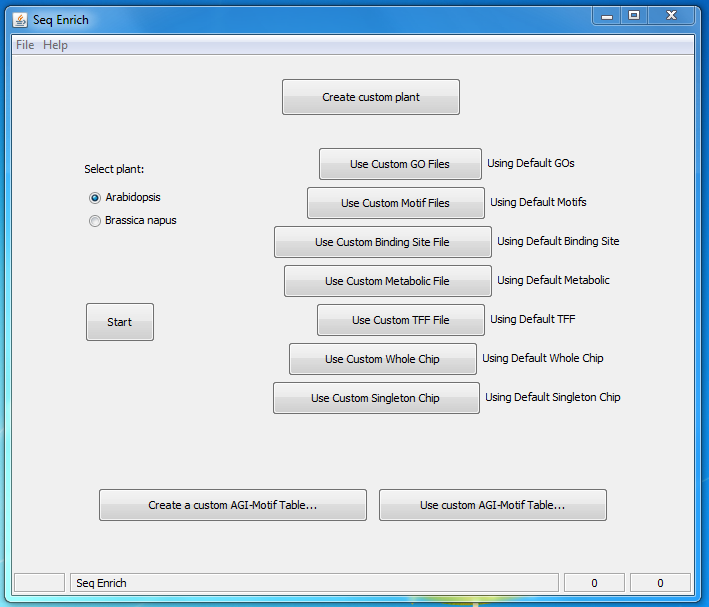
**Usage in SeqEnrich:**

SeqEnrich is distributed as a Java jar file and can be opened by double clicking the jar file or alternatively through the command line using: “Java -jar directory to SeqEnrich”. You will need Java installed on the computer. After opening the program, the user must select the organism they will be working with. Currently two options are available: Arabidopsis or *Brassica napus*.


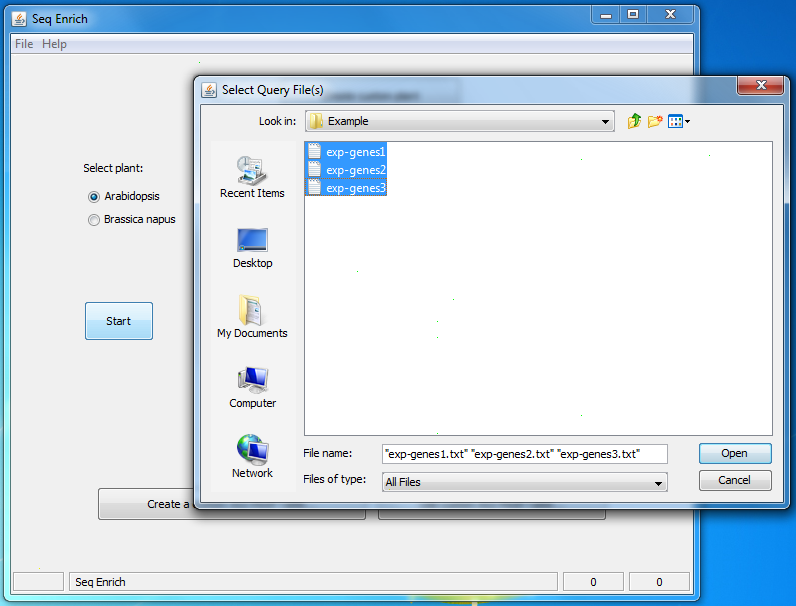
Default databases are automatically uploaded in SeqEnrich and ready to run; however, if the user has created a custom database they would rather use in their analysis, the option to upload a database is given by the ‘Use Custom ___ File’ options. If the user has uploaded a custom database, they will need to reconstitute an AGI-motif table. This can be done by selecting 'Create a custom AGI Motif-Table'. This will prompt the user for a fasta file of all promoters and subsequently prompt for a new tab delimited file of motifs. While these options exist, the program already contains all current information available on Arabidopsis and *Brassica napus cis*-regulatory elements and will be updated regularly.


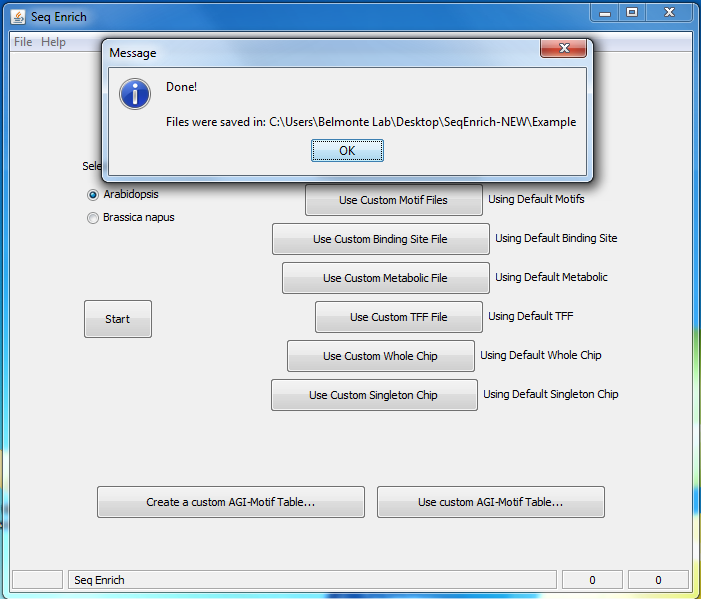
To initiate the program, the user simply needs to select the ‘Start’ button. The user will then be prompted to input any query gene list(s) to be analyzed. Gene lists should be a text file containing the accessions of either Arabidopsis (e.g. AT1G01010) or *Brassica napus* (e.g. BnaA01g00010D) genes to be tested, with one gene per line. Multiple gene lists, each from a separate file, can be analyzed in the same run by holding CTRL and selecting multiple files.

After the query lists have been selected the program will begin processing. Once complete the program will issue a notification and identify the output directory, which will be within the directory that contained the input list.


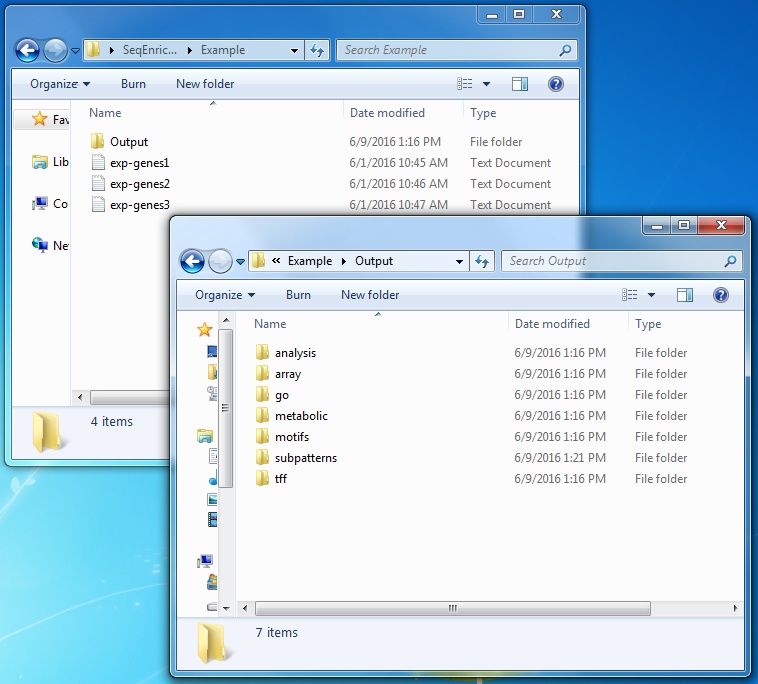
The output folder consists of seven subfolders, each containing the results from separate analyses. If the user is interested in visualizing their data by building transcription factor networks, the analysis subfolder contains all necessary files.

Each sample will have four text files associated with it;
.analysis.networks.txt
.analysis.attributes.txt


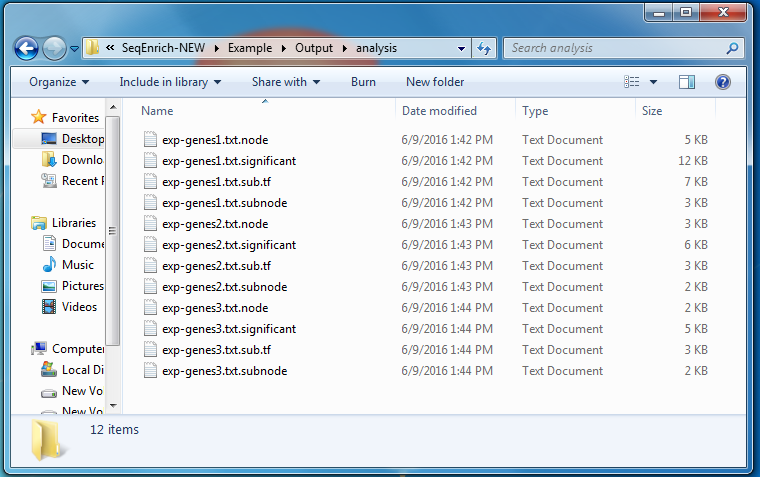
.subanalysis.networks.txt

.subanalysis.attributes.txt

The two analysis files identify GO terms and motifs enriched within the entire query list (aka. pattern) and the subanalysis files identify motifs enriched within individual enriched GO terms (aka. subpattern).

**Using Cytoscape to visualize transcription factor networks:**


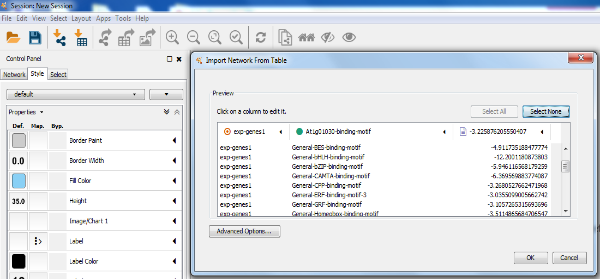

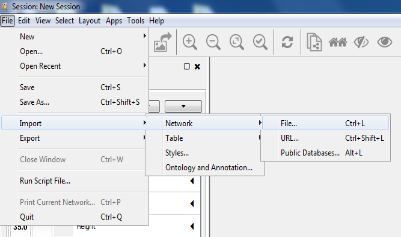
Begin by importing a network file. Go to File > Import > Network > File and open the .analysis.networks.txt or .subanalysis.networks.txt file. The program will prompt the user with a preview containing three columns. Under ‘Meaning’ set the first column to “Target Node’, second column to ‘Source Node’, and third column to ‘Edge Attribute’. If using an older version of Cytoscape, you may have to change column settings and uncheck delimiters “space” and select “tab” so that columns displayed properly.


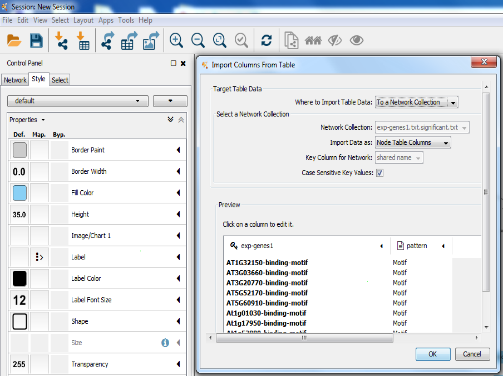

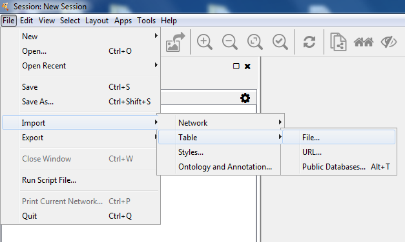
Once the network file is imported a table file (attributes) must also be imported to assign features to the nodes. Go to File > Import > Table > and open the associated .analysis.attributes.txt (pattern) or .subanalysis.attributes.txt (subpattern) and click OK. Once imported you should check to make sure all patterns are labeled correctly in the table using the table panel, if anything is not labeled or if a column is incorrectly labeled, rename it appropriately.


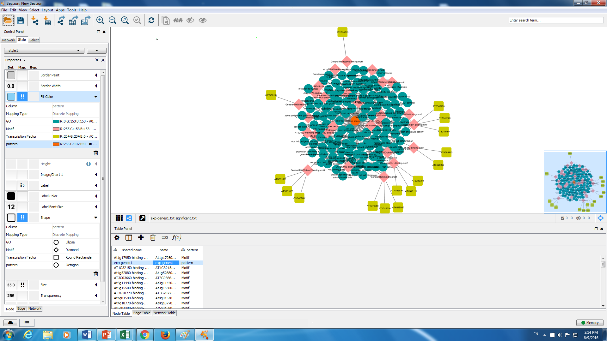

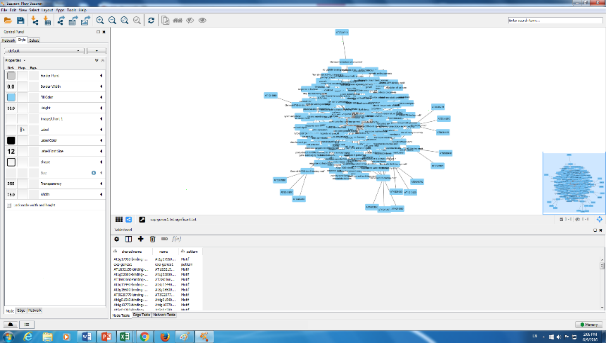
Once you have imported your files, nodes and interactions will appear in cytoscape. Using the ‘Style’ feature in the control panel, networks can be customized by changing node colors, shape, and size. Start by clicking the ‘Fill Color’ tab. Under Column, select the label that corresponds to the third column of your table panel containing each node attribute on the bottom of your screen. For Mapping Type select ‘Discrete Mapping’, and select the desired colour for each node. In our examples we use teal for the ‘GO’ nodes, peach for ‘Motif’ nodes and chartreuse for the ‘Transcription Factor’ nodes. Once you have given each node a distinct color you do the same with the shape and size tabs. If you would like to label your individual nodes select ‘Label’ in the style menu and change Mapping Type to ‘Passthrough Mapping’. This will label each node coressponding to its ID in the table file.


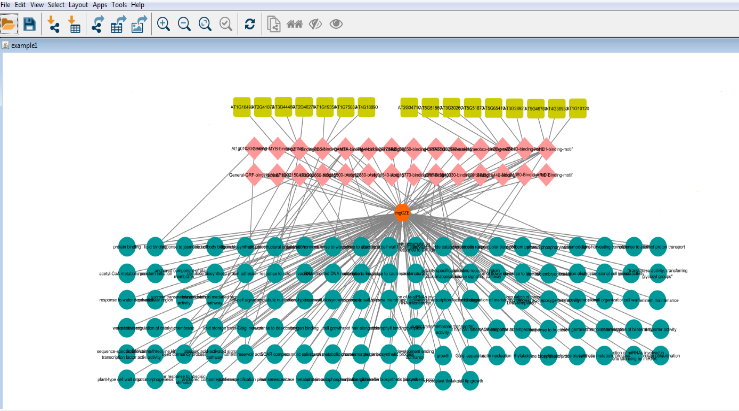


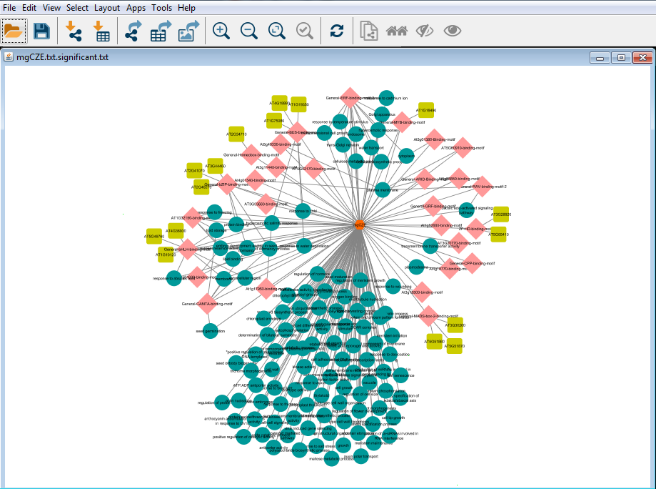
After style has been adjusted, nodes can be sorted in the workspace to make networks more intuitive. First, from the top toolbar, select Layout > Group Attributes Layout > (Label on attributes column). This will sort your network based on their attribute (ie. GOs, motifs, and transcription factors). You can further organize the network by selecting all TF’s, motif’s, or GOs (by holding CTRL and using the selection box to select all of that type), and then using Layout > Grid Layout > Selected Nodes Only. Alternatively, there are many ways to sort your networks in the Layout pulldown menu. Nodes can also be rearranged manually by dragging, and nodes/interactions can be removed by right clicking and selecting ‘cut’.


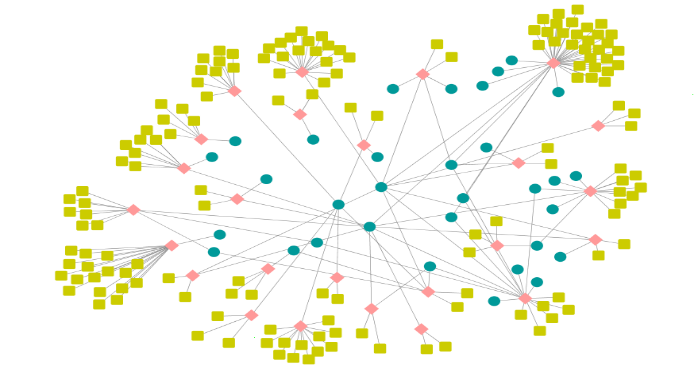
Once your network has been completed you may export the network as an image file by going to File > Export > Network View as Graphics. Images may be exported as png, jpeg or pdf files based on user preference. To ensure high quality images it is recommended that image resolution be set to the maximum (600 dpi).
