# Supplementary material for: SeqEnrich: A tool to predict transcription factor networks from co-expressed Arabidopsis and Brassica napus gene sets
Source: PLoS One. 2017 Jun 2;12(6):e0178256. doi: 10.1371/journal.pone.0178256 (PMC5456048; doi:10.1371/journal.pone.0178256)
Supplement: S2 File — Updated versions of the SeqEnrich source code will be deposited as they become available at the SourceForge open-source repository (https://sourceforge.net/). (ZIP) [file pone.0178256.s002.zip › seqenrich_src/com/bc/chipenrich/ui/about.html]

|  |
| --- |
| Chip Enrich |
| Contact: | Siobhan Brady: | sbrady@ucdavis.edu |
|  | Jeremy Koch: | jerkoch@gmail.com |
| Seq Enrich (2016) |
| Contact: | Michael Becker: | umbeck26@myumanitoba.ca |
