# Supplementary material for: SeqEnrich: A tool to predict transcription factor networks from co-expressed Arabidopsis and Brassica napus gene sets
Source: PLoS One. 2017 Jun 2;12(6):e0178256. doi: 10.1371/journal.pone.0178256 (PMC5456048; doi:10.1371/journal.pone.0178256)
Supplement: S2 File — Updated versions of the SeqEnrich source code will be deposited as they become available at the SourceForge open-source repository (https://sourceforge.net/). (ZIP) [file pone.0178256.s002.zip › seqenrich_src/org/apache/commons/logging/impl/package.html]

Concrete implementations of commons-logging wrapper APIs.
