# Supplementary material for: SeqEnrich: A tool to predict transcription factor networks from co-expressed Arabidopsis and Brassica napus gene sets
Source: PLoS One. 2017 Jun 2;12(6):e0178256. doi: 10.1371/journal.pone.0178256 (PMC5456048; doi:10.1371/journal.pone.0178256)
Supplement: S2 File — Updated versions of the SeqEnrich source code will be deposited as they become available at the SourceForge open-source repository (https://sourceforge.net/). (ZIP) [file pone.0178256.s002.zip › seqenrich_src/org/apache/commons/logging/package.html]

Simple wrapper API around multiple logging APIs.

### Overview

This package provides an API for logging in server-based applications that
can be used around a variety of different logging implementations, including
prebuilt support for the following:

- Log4J (version 1.2 or later)
  from Apache's Jakarta project. Each named Log
  instance is connected to a corresponding Log4J Logger.
- JDK Logging API, included in JDK 1.4 or later systems. Each named
  Log instance is connected to a corresponding
  `java.util.logging.Logger` instance.
- LogKit from Apache's
  Avalon project. Each named Log instance is
  connected to a corresponding LogKit `Logger`.
- NoOpLog implementation that simply swallows
  all log output, for all named Log isntances.
- SimpleLog implementation that writes all
  log output, for all named Log instances, to
  System.err.

### Quick Start Guide

For those impatient to just get on with it, the following example
illustrates the typical declaration and use of a logger that is named (by
convention) after the calling class:

```
    import org.apache.commons.logging.Log;
    import org.apache.commons.logging.LogFactory;

    public class Foo {

        static Log log = LogFactory.getLog(this.class);

        public void foo() {
            ...
            try {
                if (log.isDebugEnabled()) {
                    log.debug("About to do something to object " + name);
                }
                name.bar();
            } catch (IllegalStateException e) {
                log.error("Something bad happened to " + name, e);
            }
            ...
        }
```

Unless you configure things differently, all log output will be written
to System.err. Therefore, you really will want to review the remainder of
this page in order to understand how to configure logging for your
application.

### Configuring the Commons Logging Package

#### Choosing A `LogFactory` Implementation

From an application perspective, the first requirement is to retrieve an
object reference to the `LogFactory` instance that will be used
to create `Log` instances for this
application. This is normally accomplished by calling the static
`getFactory()` method. This method implements the following
discovery algorithm to select the name of the `LogFactory`
implementation class this application wants to use:

- Check for a system property named
  `org.apache.commons.logging.LogFactory`.
- Use the JDK 1.3 JAR Services Discovery mechanism (see
  http://java.sun.com/j2se/1.3/docs/guide/jar/jar.html for
  more information) to look for a resource named
  `META-INF/services/org.apache.commons.logging.LogFactory`
  whose first line is assumed to contain the desired class name.
- Look for a properties file named `commons-logging.properties`
  visible in the application class path, with a property named
  `org.apache.commons.logging.LogFactory` defining the
  desired implementation class name.
- Fall back to a default implementation, which is described
  further below.

If a `commons-logging.properties` file is found, all of the
properties defined there are also used to set configuration attributes on
the instantiated `LogFactory` instance.

Once an implementation class name is selected, the corresponding class is
loaded from the current Thread context class loader (if there is one), or
from the class loader that loaded the `LogFactory` class itself
otherwise. This allows a copy of `commons-logging.jar` to be
shared in a multiple class loader environment (such as a servlet container),
but still allow each web application to provide its own `LogFactory`
implementation, if it so desires. An instance of this class will then be
created, and cached per class loader.

#### The Default `LogFactory` Implementation

The Logging Package APIs include a default `LogFactory`
implementation class (
org.apache.commons.logging.impl.LogFactoryImpl) that is selected if no
other implementation class name can be discovered. Its primary purpose is
to create (as necessary) and return Log instances
in response to calls to the `getInstance()` method. The default
implementation uses the following rules:

- At most one `Log` instance of the same name will be created.
  Subsequent `getInstance()` calls to the same
  `LogFactory` instance, with the same name or `Class`
  parameter, will return the same `Log` instance.
- When a new `Log` instance must be created, the default
  `LogFactory` implementation uses the following discovery
  process is used:
  - Look for a configuration attribute of this factory named
    `org.apache.commons.logging.Log` (for backwards
    compatibility to pre-1.0 versions of this API, an attribute
    `org.apache.commons.logging.log is also consulted)..`
  - Look for a system property named
    `org.apache.commons.logging.Log` (for backwards
    compatibility to pre-1.0 versions of this API, a system property
    `org.apache.commons.logging.log` is also consulted).
  - If the Log4J logging system is available in the application
    class path, use the corresponding wrapper class
    (Log4JLogger).
  - If the application is executing on a JDK 1.4 system, use
    the corresponding wrapper class
    (Jdk14Logger).
  - Fall back to the default simple logging wrapper
    (SimpleLog).
- Load the class of the specified name from the thread context class
  loader (if any), or from the class loader that loaded the
  `LogFactory` class otherwise.
- Instantiate an instance of the selected `Log`
  implementation class, passing the specified name as the single
  argument to its constructor.

See the SimpleLog JavaDocs for detailed
configuration information for this default implementation.

#### Configuring the Underlying Logging System

The basic principle is that the user is totally responsible for the
configuration of the underlying logging system.
Commons-logging should not change the existing configuration.

Each individual Log implementation may
support its own configuration properties. These will be documented in the
class descriptions for the corresponding implementation class.

Finally, some `Log` implementations (such as the one for Log4J)
require an external configuration file for the entire logging environment.
This file should be prepared in a manner that is specific to the actual logging
technology being used.

### Using the Logging Package APIs

Use of the Logging Package APIs, from the perspective of an application
component, consists of the following steps:

1. Acquire a reference to an instance of
   org.apache.commons.logging.Log, by calling the
   factory method
   LogFactory.getInstance(String name). Your application can contain
   references to multiple loggers that are used for different
   purposes. A typical scenario for a server application is to have each
   major component of the server use its own Log instance.
2. Cause messages to be logged (if the corresponding detail level is enabled)
   by calling appropriate methods (`debug()`, `info()`,
   `warn()`, `error`, and `fatal()`).

For convenience, `LogFactory` also offers a static method
`getLog()` that combines the typical two-step pattern:

```
  Log log = LogFactory.getFactory().getInstance("Foo");
```

into a single method call:

```
  Log log = LogFactory.getLog("Foo");
```

For example, you might use the following technique to initialize and
use a Log instance in an application component:

```
import org.apache.commons.logging.Log;
import org.apache.commons.logging.LogFactory;

public class MyComponent {

  protected static Log log =
    LogFactory.getLog("my.component");

  // Called once at startup time
  public void start() {
    ...
    log.info("MyComponent started");
    ...
  }

  // Called once at shutdown time
  public void stop() {
    ...
    log.info("MyComponent stopped");
    ...
  }

  // Called repeatedly to process a particular argument value
  // which you want logged if debugging is enabled
  public void process(String value) {
    ...
    // Do the string concatenation only if logging is enabled
    if (log.isDebugEnabled())
      log.debug("MyComponent processing " + value);
    ...
  }

}
```
